# Editorial: Computing and artificial intelligence in digital therapeutics

**DOI:** 10.3389/fmed.2023.1330686

**Published:** 2024-01-05

**Authors:** Pengwei Hu, Lun Hu, Fei Wang, Jing Mei

**Affiliations:** ^1^The Xinjiang Technical Institute of Physics and Chemistry, Chinese Academy of Sciences, Ürümqi, China; ^2^Department of Population Health Sciences, Weill Cornell Medicine, Cornell University, New York, NY, United States; ^3^Ping An Technology, Shenzhen, China

**Keywords:** digital therapeutics, artificial intelligence, machine learning, digital health, medical informatics

## 1 Intelligent digital therapeutics: future outlook

As technology continues to advance, the field of digital therapeutics(DTx) has garnered increasing attention (1). DTx is a novel concept that uses computing and medical devices to prevent, manage, and treat diseases. It offers features such as convenience, personalization, and efficiency, leading to its widespread application in the healthcare sector (2). With the development of computing and hardware technologies, the scope of DTx is expanding, benefiting a greater number of patients. For example, the integration of wearable devices allows for more precise treatment, enabling each patient to receive a tailored treatment plan (3). The history of DTx can be traced back to the 1960s when the first prototype of DTx, ELIZA (4), a virtual psychotherapist based on a dialogue system, was created. During this period, computer technology was becoming more widespread, and researchers began to explore how to utilize it for healthcare services. At this stage, DTx primarily relied on simple software and hardware devices, using expert systems to achieve some intelligent functions but lacking advanced computing technology and intelligent algorithms. With the continuous development of computer technology and network technology, DTx gradually received more attention and applications. Researchers started to explore how to enhance the therapeutic effectiveness and efficiency of DTx using advanced computing technology and intelligent algorithms. During this phase, DTx evolved into an independent field within healthcare, attracting more researchers and companies to participate (5).

Around 2017, the FDA began to approve disease intervention App as certified DTx products (6). By 2020, the global DTx market had flourished, and DTx products began to benefit from expedited approval processes and rapid market access dozens of DTx products obtained fast approvals and certifications within just a year. In summary, the development of DTx has been a continuous process of exploration, innovation, and standardization. In the future, with ongoing technological advancements and policy improvements, DTx will play an increasingly vital role in healthcare (7). Moreover, the DTx field is experiencing explosive growth, with a continuous influx of various DTx products covering a wider range of medical conditions. This not only meets the needs of diverse patient groups and improves treatment outcomes but also brings new opportunities and challenges to the healthcare sector. Compared to early DTx products, modern DTx has gradually introduced artificial intelligence algorithms. Currently, artificial intelligence algorithms are primarily applied in high-throughput scenarios such as disease screening and prevention. However, to fully leverage artificial intelligence in the finely managed field of digital healthcare, more research resources have been invested in this area (8). The combination of artificial intelligence and DTx can further enhance the effectiveness and efficiency of DTx. Multiple studies have shown that by utilizing artificial intelligence algorithms to analyze and mine extensive patient data, more personalized treatment plans can be formulated, leading to improved treatment outcomes (9, 10). However, the application of artificial intelligence in DTx also faces several challenges. Firstly, the accuracy and reliability of artificial intelligence algorithms need further validation and confirmation. Secondly, the application of artificial intelligence requires substantial data support, and acquiring and processing this data pose significant challenges. The development of DTx not only relies on technological support but also necessitates close integration with clinical research (11). While past digital healthcare primarily focused on health management, the future is centered on therapeutic functionalities. This requires conducting comprehensive clinical research for each new DTx product to validate its safety and efficacy. In clinical research, relevant norms and standards must be established to ensure the reliability and reproducibility of studies. Clinical experience also needs to be better summarized and used as a reference to help healthcare providers and patients understand and apply DTx effectively. Additionally, the application of artificial intelligence must consider privacy protection and ethical issues (12). Therefore, in the future development of DTx, there is a need to further strengthen technological research and policy development to standardize the research and application of DTx. Simultaneously, there is a need to enhance societal awareness and increase public trust and acceptance of DTx. We believe that DTx integrated with artificial intelligence and computing technology will become the industry standard of the future. DTx will transition from the era of DTx to the era of intelligent DTx (iDTx), and the future will witness extensive research on the intersection of digital therapy and intelligent technology. With breakthroughs in hardware technology, iDTx will benefit patients even more.

## 2 Policy, regulations, and consensus support the deployment of digital therapeutics

The time that digital therapeutics have truly entered human life is only a few short years. As a new phenomenon, policy makers, policy implementers, physicians, patients, and communities are all learning to accept it. Participants, starting from their respective roles, are actively exploring how to use digital therapeutics correctly. Lutz et al. delved into the intricacies surrounding the implementation of appropriate controls for digital therapeutics (DTx) within the context of clinical trials. The discourse primarily centered on critical factors pertaining to the design and regulatory facets of digital therapeutics, underscoring the imperative for meticulous evidence substantiation through rigorous clinical trials. Additionally, the article brought into focus the inherent potential of real-world data aggregation as a pivotal means to evaluate the efficacy and user engagement of DTx. The study accentuated the paramount significance of randomized controlled trials (RCTs) in furnishing the requisite evidentiary foundation for DTx, particularly those poised for regulatory endorsement. It was posited that the rigidity of digital control conditions could be contingent upon variables such as the risk profile and innovative nature of the intervention. Furthermore, the article posited that, given the generally subdued risk profile associated with DTx, there exists a conceivable propensity toward the adoption of less stringent controls, or even the incorporation of waitlist controls, as a strategic measure to enhance accessibility to personalized care. Noteworthy attention was also directed toward the potential hazards entailed in DTx, encompassing technical exigencies and the conceivable inadequacy in treatment selection. Consequently, the authors advocated for a thoroughgoing integration of these considerations in forthcoming DTx trials. Moreover, the article judiciously acknowledged the prevailing US-centric orientation of the majority of the trials scrutinized, thereby, conscientiously apprising that the regulatory deliberations proffered may not comprehensively encompass potential divergences in regulatory protocols beyond the purview of the United States. The authors astutely underscored the unique capacity of DTx to directly amass real-world data through their software applications, thereby, lending substantial support to the burgeoning traction of real-world and pragmatic study methodologies in the realm of evidence generation.

Tong et al. explored the concept of Digital Therapeutic Alliance (DTA) with fully automated apps. It delved into the significance of the therapeutic alliance in the context of digital mental health interventions, specifically those involving automated applications. The study emphasized the importance of the user's emotional attachment and trust in these applications, drawing from theories of attachment in human relationships. The authors discussed various aspects related to DTA, including its role in internet interventions, the impact on outcomes, and comparisons between face-to-face and digital treatments. The article also provided insights into the psychometrics of the Working Alliance Inventory for Virtual and Augmented Reality (WAI-VAR) and its relevance to digital interventions. Furthermore, the article touched on factors influencing DTA, such as the design and functionality of mental health apps, as well as the user experience and adherence to these interventions. The article highlighted the potential of technology, particularly in establishing a sense of autonomy, control, and attachment for users. Overall, the article underscored the evolving landscape of therapeutic relationships in the digital realm, shedding light on the multifaceted dynamics between users and automated mental health applications. It aimed to contribute to the ongoing discourse surrounding the integration of technology in mental health care.

Gu et al. explored the intersection of healthcare, technology, and innovation in China. The study emphasized the importance of technological innovation in healthcare organizations, particularly in the context of digital health. It highlighted the correlation between advancements in digital health and regional economic development, suggesting that areas with better economic conditions tended to have more innovative healthcare systems. The article introduced an evaluation system for medical institutions, focusing on indicators like the number of patents matched per article, the number of articles matched per patent, and the proportion of highly matched patents and articles. These metrics provided a novel perspective on evaluating the relationship between scientific research and technological innovation in hospitals, which was previously overlooked by existing ranking systems. The study emphasized the role of healthcare workers in driving innovation in the digital health field, underscoring the importance of their contributions. It also touched upon the limitations of evaluating hospital performance solely based on size, suggesting that it may not necessarily lead to increased innovative capacity. This article makes significant contributions to DTx by introducing a novel evaluation system for healthcare institutions, emphasizing the critical role of technological innovation. It highlights the correlation between digital health advancements and regional economic development, offering valuable insights for policymakers and stakeholders in the DTx space. Additionally, the study underscores the pivotal contribution of healthcare workers in driving innovation, emphasizing the need for their active involvement in the development of digital health solutions.

Markatou et al. discussed various aspects related to social determinants of health. It covered a range of topics, including the impact of drug addiction stigma on methadone maintenance therapy, reforming physician payments for enhanced equity and value in healthcare, and strategies for screening social determinants of health in populations with complex needs. It highlighted the challenges associated with collecting, integrating, and effectively using clinical data for this purpose. The authors proposed a collaborative approach, bringing together expertise from medical, statistical, and computer and data science domains. They emphasized the importance of using provenance-aware, self-documenting workflow tools to facilitate data integration and create reproducible workflows. By addressing the unique data needs of underserved populations, this approach aimed to improve well-being and drive policy improvements for individuals with OUD. The article concludes by advocating for policy frameworks to address disparities and discrimination in healthcare. Overall, it offers a comprehensive overview of factors influencing health outcomes, from social determinants to policy considerations. Understanding the adoption of computing and AI technologies among DTx users will also be critical to guide product developers and policy makers.

## 3 Emerging advanced models illuminate the development path of intelligent digital therapeutics

Upon comprehensive examination of prevailing advanced models, it is evident that machine learning technology has attained a heightened level of refinement, thereby accruing significant technical acumen within the medical domain. Nevertheless, digital healthcare constitutes a complex and multidisciplinary domain necessitating profound scrutiny when implementing models, with the overarching objective of attaining optimal alignment with the patient's therapeutic journey. This affords robust tools for physician management and affords patients precision in diagnosis and treatment, potentially augmenting the degree of human-machine interactivity. Pan et al. focused on developing a risk prediction model for Type 2 diabetes. The study explored various factors associated with diabetic retinopathy, a complication of diabetes that affects the eyes. It incorporated a wide range of data, including clinical parameters, demographic information, and medical history, to build a comprehensive predictive model. The article extensively reviewed existing literature and studies related to diabetes, particularly diabetic retinopathy. It also incorporated machine learning techniques and advanced statistical models to enhance the accuracy of risk prediction. The research considered a diverse set of variables, such as blood pressure, kidney function, and glycemic control, to effectively predict the likelihood of diabetic retinopathy in individuals with Type 2 diabetes. This article makes a significant contribution to DTx by introducing a robust risk prediction model for Type 2 diabetes. This model provides a valuable tool for digital platforms to implement proactive interventions, allowing for early detection and personalized management of diabetic retinopathy in individuals with Type 2 diabetes. The prediction of diseases constitutes a foundational domain within AI in healthcare. Consequently, research in this field is progressing toward more intricate disease prediction and encompassing larger-scale data modeling.

Kong et al. outlined an intelligent diabetes big data processing and analysis system designed to handle diverse and extensive medical data related to diabetes. It employed advanced technologies like Hadoop Distributed File System (HDFS) and Hadoop Database (HBase) for distributed data storage and processing, accommodating the inherent complexities of medical data. The system integrated data mining algorithms, including XGBoost, LightGBM, and K-Nearest Neighbor, for tasks such as missing value handling and disease prediction. Emphasis was placed on data security and privacy, utilizing blockchain and privacy computing techniques. A user-friendly data visualization interface offered statistical analysis through methods like heat maps and word clouds. The system was modular, encompassing scientific research data support, data governance, analysis, visualization, intelligent follow-up, and system management. The authors stressed its potential in improving patient care, early disease detection, and precision medicine, underlining the need for ongoing research and development to enhance its performance and expand its capabilities to cover other disease-related functions.

Wang et al. developed a novel conversational agent known as the STEF agent, designed with a focus on historical support strategies and the integration of the user's mental state. The authors proposed the use of a strategy tendency encoder to capture the trends in support strategies and an emotional fusion mechanism to incorporate the influence of past mental states. The experiments and analyses conducted demonstrated that the STEF agent showed promising performance in generating supportive responses. However, it noted a lack of response diversity, indicating room for improvement. The article also outlined certain limitations, such as the need for annotated support strategy data and the necessity for more diverse strategies in each phase. Future work was suggested to address these limitations and enhance the STEF agent. The study concluded by emphasizing the potential of the STEF agent in providing personalized responses in digital therapeutic solutions. It also discussed possibilities for further enhancements, including utilizing recorded dialog, incorporating more professional counseling skills, and implementing multilingual support. The use of dialogue systems as virtual assistants in DTx has proven effective, and various studies are beginning to explore the role of dialogue technology in a more important role.

Burger et al. discussed a study that explored the feasibility of using a conversational agent for thought recording as a cognitive therapy task, particularly for individuals with subclinical depression symptoms. The study involved participants completing thought records using a conversational agent, and the results indicated that this approach was viable, with all participants successfully completing the task. The research also examined the impact of feedback richness on motivation and engagement. The findings showed that feedback richness did not significantly influence motivation, but participants who reported a greater need for self-reflection reported higher engagement in the task. Additionally, the study provided insights into the content and frequency of the thought records, highlighting that participants often focused on interpersonal and social situations. The document concluded by suggesting potential avenues for future research, including combining content-based feedback with motivational interviewing strategies. Overall, the study suggested that using a conversational agent for thought recording could be a valuable tool in cognitive therapy, especially for individuals with subclinical depression symptoms.

## 4 Validation and observation are crucial factors for the success of DTx

The pivotal advancement from digital health to DTx hinges on the comprehensive validation of digital therapeutic modalities, thereby heightening our expectations of digital health to a level equivalent to clinical medicine. Similarly, the acceptance of AI technology in clinical practice has been achieved through a succession of rigorous clinical trials, fostering a willingness to entrust patient diagnoses to AI. Consequently, to judiciously incorporate AI in DTx and to engender conviction among both healthcare practitioners and patients, substantial research endeavors are requisite. This necessitates an augmented scrutiny and validation of DTx from diverse vantage points.

Chen et al. provided a series of studies and surveys delving into the acceptance and perception of clinical artificial intelligence (AI) within the medical community. It drew from a diverse array of research articles and surveys spanning different regions and medical specialties. These investigations scrutinized the viewpoints, concerns, and inclinations of healthcare professionals, including physicians, radiologists, dermatologists, and medical students, regarding the integration of AI in clinical workflows. They explored pivotal factors such as AI's perceived impact on medical professions, willingness to embrace AI technologies, and the potential benefits and hurdles linked with their adoption. Furthermore, the document shed light on the imperative of tailored education to address any reservations pertaining to AI integration, underlining the significance of relevant training in facilitating seamless AI assimilation and alleviating potential apprehensions among healthcare professionals. Additionally, it touched upon aspects of data accessibility for research transparency and ethical considerations, emphasizing the necessity for approval from research ethics committees in studies involving AI in healthcare. This compilation offered valuable insights into how healthcare professionals across diverse disciplines and regions engaged with and perceived clinical artificial intelligence, ultimately contributing to a more seamless integration of DTx into clinical practice.

Chalutz Ben-Gal investigated the factors that influenced individuals' readiness to use Artificial Intelligence (AI)-based applications in the context of primary care (PC) during the COVID-19 pandemic. The study employed the Technology Readiness and Acceptance Model (TRAM) to investigate patients' perspectives on AI adoption. The TRAM model considered various factors like motives, professionalism, proneness to technology use, privacy concerns, empathy, and health awareness in predicting readiness to use AI applications. The findings highlighted that motives, professionalism, technology use propensity, and privacy concerns positively influenced the readiness to use AI in PC. This suggested that individuals who were more comfortable with technology and perceived it as professional and private were more likely to adopt AI-based applications in primary care. However, factors like empathy and health awareness were not significant predictors. The study concluded by emphasizing the importance of understanding these behavioral determinants for the successful integration of AI in public health care and primary care management. It also suggested that policy-makers and health institutions should consider adaptive, population-specific promotions of AI technologies to enhance their acceptance and usability.

Nguyen et al. utilized smartphone data to track various behavioral and psychological indicators associated with anxiety levels. The researchers proposed a method to classify anxiety severity based on this data, offering a potential tool for population-level mental health assessment. The study also emphasized the importance of reducing survey fatigue in data collection and suggested augmenting passive sensor data with traditional self-reporting. The authors proved that the proposed ideas through a series of empirical studies. The authors first collected a substantial amount of data from smartphones, including behavioral and psychological indicators, as well as information related to anxiety levels. They then used this data to conduct analyses and experiments to establish the correlation with the severity of anxiety. The authors highlighted the relevance of their work in the context of the ongoing pandemic and its potential for informing public health policies.

Shetty et al. investigated the landscape of digital health interventions tailored for chronic pain management. By synthesizing a diverse range of studies, it explored the effectiveness of various digital tools, including mobile applications and online self-help programs, in assisting individuals dealing with persistent pain conditions. The review encompassed a wide spectrum of pain disorders, such as chronic headaches, fibromyalgia, and arthritis. The collective findings suggested that digital health technologies held significant promise in alleviating chronic pain, ultimately leading to an enhanced quality of life and empowering patients in self-managing their condition. However, the study highlighted the importance of personalized treatment approaches and the necessity for robust assessments of their effectiveness. In essence, this review underscored the potential of digital health technologies in chronic pain management, while emphasizing the ongoing need for research and refinement in this field.

Muto et al. proposed a predictive artificial intelligence (AI) model to assist in clinical decision-making for patients with COVID-19. The authors compared the performance of AI alone vs. AI in collaboration with a clinician to predict the need for supplemental oxygen. The study enrolled 30 elderly patients with COVID-19 and found that the AI-clinician model outperformed AI alone. Additionally, the study identified a novel indicator, sodium chloride difference, as a predictor of oxygen requirement. The authors suggested that this model, which incorporated clinician feedback, could be useful in guiding treatment decisions and improving patient outcomes in COVID-19 and other healthcare scenarios. The integration of clinician feedback strengthens the model's performance, highlighting the promising synergy between AI technology and medical expertise in DTx applications.

Zhang et al. investigated the effectiveness of a smartphone-based digital therapeutics (DTx) application in addressing methamphetamine use disorder (MUD) within a community setting. One hundred participants were randomly assigned to either the DTx group or the treatment as usual (TAU) group. Over eight weeks, the DTx group received a combination of cognitive behavioral therapy, approach bias modification, cognitive training, and contingency management through the smartphone application, while the TAU group received counseling from professionals. Results indicated significant reductions in drug craving and enhancements in cognitive function within the DTx group. The findings suggest that DTx could serve as a valuable adjunct to community-based substance use treatment programs for MUD.

## 5 Discussion and conclusion

To summarize, we have traversed the landscape of digital therapeutics (DTx), tracing its evolution from rudimentary software to a sophisticated domain seamlessly integrated with artificial intelligence. The combination of wearable technology and advanced computing has opened up a new era of personalized treatment plans, significantly enhancing the effectiveness of DTx. Importantly, robust policy frameworks and rigorous clinical trials have emerged as key factors in gaining trust among healthcare practitioners and patients for the integration of AI technologies into DTx. Emerging models, driven by machine learning, demonstrate the potential to revolutionize medical practices, with risk prediction models for conditions like Type 2 diabetes and intelligent data processing systems showing significant progress. Understanding the behavioral determinants influencing the acceptance of AI-based applications in primary care is crucial for successful integration, considering factors like motives, professionalism, and privacy concerns. Additionally, utilizing smartphone data to track behavioral and psychological indicators associated with anxiety levels presents a promising approach for population-level mental health assessment. The landscape of digital health interventions tailored for chronic pain management holds great promise, offering individuals with persistent pain conditions an enhanced quality of life through self-management. The collaborative synergy of artificial intelligence and clinical expertise, as seen in predictive AI models for patients with COVID-19, highlights the potential for technology and medical expertise to seamlessly come together in DTx applications. These advances point toward a future where DTx is poised to play an increasingly pivotal role in healthcare, benefiting a diverse array of patient groups and paving the way for an era of intelligent, personalized medical solutions.

## Author contributions

PH: Writing—original draft, Writing—review & editing. LH: Writing—original draft, Writing—review & editing. FW: Writing—original draft, Writing—review & editing. JM: Writing—original draft, Writing—review & editing.

## References

[1] Makin S. The emerging world of digital therapeutics. Nature. (2019) 573:S106. 10.1038/d41586-019-02873-1 31554990

[2] Dang A Arora D Rane P. Role of digital therapeutics and the changing future of healthcare. J Fam Med Prim Care. (2020) 9:2207. 10.4103/jfmpc.jfmpc_105_20 32754475 PMC7380804

[3] Joshi S Verma R Lathia T Selvan C Tanna S Saraf A. Fitterfly Diabetes CGM Digital therapeutics program for glycemic control and weight management in people with type 2 diabetes mellitus: real-world effectiveness evaluation. JMIR Diabetes. (2023) 8:e43292. 10.2196/43292 37133922 PMC10193208

[4] Weizenbaum J. ELIZA a computer program for the study of natural language communication between man and machine. Commun ACM. (1966) 9:36–45. 10.1145/365153.365168

[5] Wang C Lee C Shin H. Digital therapeutics from bench to bedside. NPJ Digit Med. (2023) 6:38. 10.1038/s41746-023-00777-z 36899073 PMC10006069

[6] Waltz E. FDA approves a prescription-only app for addiction [news]. IEEE Spectrum. (2017) 54:9–10. 10.1109/MSPEC.2017.8093786

[7] Crisafulli S Santoro E Recchia G Trifirò G. Digital therapeutics in perspective: from regulatory challenges to post-marketing surveillance. Front Drug Saf Regul. (2022) 2:900946. 10.3389/fdsfr.2022.900946

[8] Palanica A Docktor MJ Lieberman M Fossat Y. The need for artificial intelligence in digital therapeutics. Digit Biomark. (2020) 4:21–5. 10.1159/000506861 32399513 PMC7204774

[9] Lin C Hu P Su H Li S Mei J Zhou J. Sensemood: depression detection on social media. In: Proceedings of the 2020 International Conference on Multimedia Retrieval. New York, NY: ACM (2020), p. 407–11. 10.1145/3372278.3391932

[10] Wang Y Ma J Hao B Hu P Wang X Mei J. Automatic depression detection via facial expressions using multiple instance learning. In: 2020 IEEE 17th International Symposium on Biomedical Imaging (ISBI). Iowa City, IA: IEEE (2020). p. 1933–6. 10.1109/ISBI45749.2020.9098396

[11] Patel NA Butte AJ. Characteristics and challenges of the clinical pipeline of digital therapeutics. NPJ Digit Med. (2020) 3:159. 10.1038/s41746-020-00370-8 33311567 PMC7733514

[12] Refolo P Sacchini D Raimondi C Spagnolo AG. Ethics of digital therapeutics (DTx). Eur Rev Med Pharmacol Sci. (2022) 26:6418–23. 10.26355/eurrev_202209_29741 36196692

